# Endothelial-to-Mesenchymal Transition as Underlying Mechanism for the Formation of Double-Chambered Right Ventricle

**DOI:** 10.1007/s00246-022-02828-w

**Published:** 2022-01-27

**Authors:** Viktoria Weixler, Peter Kramer, Judith Lindner, Peter Murin, Mi-Young Cho, Pedro del Nido, Joachim Photiadis, Ingeborg Friehs

**Affiliations:** 1 Department of Congenital Heart Surgery/Pediatric Heart Surgery, German Heart Center Berlin, Berlin, Germany; 2 Department of Congenital Heart Disease/Pediatric Cardiology, German Heart Center Berlin, Berlin, Germany; 3 Institute of Pathology, Charité, Berlin, Germany; 4 Department of Cardiac Surgery, Harvard Medical School, Boston Children’s Hospital, 300 Longwood Ave, Boston, MA 02115 USA

**Keywords:** Endothelial-to-mesenchymal transition, Double-chambered right ventricle

## Abstract

Double-chambered right ventricle (DCRV) is a progressive division of the right ventricular outflow tract (RVOT) often associated with a subaortic ventricular defect (VSD). The septation is caused by a mixture of hypertrophied muscle bundles and fibrous tissue, whereof the latter is of unclear pathogenesis. Our group has previously reported that flow disturbances lead to formation of fibroelastic tissue through a process called endothelial-to-mesenchymal transition (EndMT) but it is unclear whether the same mechanism exists in the RV. Tissue from patients undergoing repair of DCRV was examined to identify the histomorphological substrate of this tissue. Demographic and pre-/post-operative echocardiographic data were collected from nine patients undergoing surgery for DCRV. RVOTO tissue samples were histologically analyzed for myocardial hypertrophy, fibrosis, elastin content, and active EndMT (immunohistochemical double-staining for endothelial and mesenchymal markers and transcription factors Slug/Snail) and compared to four healthy controls. Indication for surgery were symptoms and progressive RVOT gradients. A highly turbulent flow jet through the RVOTO and VSD was observed in all patients with a preoperative median RVOT peak gradient of 77 mmHg (IQR 55.0–91.5), improved to 6 mmHg (IQR 4.5–17) postoperatively. Histological analysis revealed muscle and thick infiltratively growing fibroelastic tissue. EndMT was confirmed as underlying patho-mechanism of this fibroelastic tissue but the degree of myocardial hypertrophy was not different compared to controls (*P* = 0.08). This study shows for the first time that an invasive fibroelastic remodeling processes of the endocardium into the underlying myocardium through activation of EndMT contributes to the septation of the RVOT.

## Introduction

Double-chambered right ventricle (DCRV) is a rare congenital heart condition, found in less than 3% of all congenital heart defects and is detected in 1:36,000 autopsies [1]. First described in 1858 by Peacock as a mid-cavitary right ventricular outflow tract obstruction (RVOTO), it is defined as a progressive division of the right ventricle (RV) into two chambers, a high-pressure inlet- and a low-pressure outlet chamber [1–3]. A subaortic ventricular septal defect (VSD) is the most common associated anomaly occurring in > 90% of all patients [4].

Although the exact genesis of this pathological RV septation is still unknown, several mechanisms have been proposed including hypertrophy of the moderator band and/or hypertrophy of accessory septo-parietal trabeculations [1]. Since surgical correction is generally performed early in life, the natural history and progression of this disease have not been thoroughly investigated. Case reports describing the formation of DCRV in adult unoperated patients suggest an important contribution of hypertrophied anomalous muscle bundles becoming obstructive over time which suggests an acquired obstruction with an underlying congenital substrate [2, 4]. Increasing intraventricular gradients measured over time in unoperated patients with DCRV support this hypothesis. Formation of two chambers within the RV, and the presence of a gradient across the RV cavity is indicative of DCRV.

Histological analyses of resected DCRV tissue describe a mixture of hypertrophied muscle bundles and fibrous tissue, whereof the latter is of unclear pathogenesis [4, 5]. One hypothesis suggests that flow disturbances play a role in the development of this fibrotic tissue. Due to the septation within the RV, blood flow must pass within the RV either above the septation, between the septation and the tricuspid valve or through a narrow channel between the septation and the septal wall which causes significant turbulence. Furthermore, increased blood flow with high-pressure jet formation is present across the VSD [6]. It has also been reported that in some cases this high-pressure flow situation may lead to spontaneous closure of the VSD over time [1, 7]. These findings indicate that the formation of DCRV is the pathophysiological result of flow disturbances rather than only hypertrophied muscle bundles that lead to the formation of DCRV.

Our group has previously reported that flow disturbances caused by jets due to valvar defects on the left side of the heart, lead to remodeling of the LV cavity through formation of fibroelastic tissue [8, 9]. Furthermore, we established that the underlying patho-mechanism for the formation of this fibroelastic tissue is endothelial-to-mesenchymal-transition (EndMT) which serves as physiological process for valve and septal formation during fetal development [10, 11]. It is, however, unclear whether the formation of fibroelastic tissue is also triggered by the same mechanism on the right side of the heart. With access to tissue resected from patients undergoing repair of DCRV, we sought to thoroughly describe the tissue histologically, and determine whether formation of fibroelastic tissue through activation of EndMT and not only hypertrophy of anomalous muscle bundles is associated with the septation of the RVOT into a two-chambered ventricle.

## Material and Methods

### Ethics Statement

The research protocol (EA2/152/19) was approved and the study conducted in accordance with the standards of the institutional ethics committee of the Charité Berlin, Germany. Patients diagnosed with DCRV scheduled to undergo surgery for RVOTO resection and/or their legal guardians were asked for consent prior to surgery and enrolled in this study. Unidentified RV histological slides from healthy hearts served as controls (*n* = 4).

### Patients

Nine patients diagnosed with DCRV who underwent RVOTO resection at the German Heart Center Berlin between September 2019 and January 2021 were included in this study. Diagnosis, medical history, previous procedures/interventions, clinical presentation prior to surgery, indication for surgery, intraoperative findings and imaging studies preoperatively and at last follow-up were recorded for all patients. During routine surgery, RVOTO tissue resected for correction of DCRV was obtained for further analysis. If a VSD was present, VSD closure with an autologous pericardial patch was performed.

### Echocardiographic Diagnosis and Assessment of Flow Disturbances

All available pre- and postoperative echocardiograms were evaluated; and all patients had at least one pre- and one postoperative echocardiogram performed at our institution and three patients underwent additional imaging studies. VSD morphology (defect size and localization), shunt direction across the VSD, and pulmonary valve morphology were assessed. Additional parameters recorded on echocardiograms included peak velocities and derived pressure gradients across the VSD and across the RVOT/PV.

### Histological Analysis of Resected RVOT Tissue

The resected tissue was stored in 10% neutral buffered formalin, embedded in paraffin and sectioned for staining and histological analysis. Tissue morphology and myocardial hypertrophy in DCRV and healthy control samples were determined by staining with hematoxylin and eosin (H&E). The number of nuclei per field of vision was used to semi-quantitatively determine the degree of myocardial hypertrophy through assessing the area of myocardium/nuclei per field of vision. Ten randomly selected fields from each slide (*n* = 9—RVOTO and *n* = 4—non-RVOTO) were taken, and analysis was performed with ImageJ (version 2.0.0-rc-43, National Institute of Health, Bethesda, MD, USA). Masson’s trichrome (MT) staining served to visualize the degree of fibrosis, and Van Giessen elastin staining to determine the involvement of elastin deposition in the disease process. Methods of quantifying muscle tissue in histologic sections have previously been described in detail by our group [12].

Active EndMT was determined by immunohistochemical identification of endothelial cells double-stained with an endothelial marker, cluster of differentiation 31 (CD31; 1:100; Dako/Agilent, Santa Clara, CA, USA), and a mesenchymal marker, α-smooth muscle actin (α-SMA; 1:100; Abcam, Cambridge, MA, USA), which is indicative of active EndMT as we have described in more detail previously [8]. In addition, all samples were stained for the transcription factors Slug/Snail (1:100; Abcam) to confirm active EndMT. Co-localization of Slug/Snail with nuclei of endothelial cells is considered affirmative of active EndMT. All slides were visualized using a Zeiss Axio Observer Z1 light microscope and fluorescence microscope (Carl Zeiss Microscopy, LLC, White Plains, NY) with a Nikon 20 × objective (numerical aperture = 20*x*/0.45, Nikon, Tokyo, Japan).

### Statistical Analysis

Descriptive data are expressed as median and interquartile range (IQR) or frequency (%). Quantitative data with normal distribution is reported as mean ± standard deviation. Comparison of DCRV samples with non-RVOTO control samples was performed using *t*-test, *P* ≤ 0.05 was considered statistically significant.

## Results

### Patients

A total of nine patients were included in this analysis and their baseline characteristics are summarized in Table 1. Male gender was dominant (66.7%; 6/9). Median age in the entire cohort was 0.8 (IQR 0.5–5.2) years with almost half of the patients (44.4%; 4/9) beyond one year of age and one adult patients. Transcutaneous oxygen saturations were within normal ranges between 96 and 99% but one patient (patient #2) displayed hypoxemia.

**Table 1:** Baseline characteristics and demographics of the patient cohort

Patient	Gender (m/f)	Age (years)	BSA at surgery (m^2^)	Preop. saturation (%)	Diagnosis	Additional anomalies	Current procedure	Previous surgeries/interventions	Symptoms/indication for surgery	First diagnosed	Prior medication
1	m	1.4	0.5	99	DCRV, VSD, RVOTO	LVOTO	RVOTO/LVOTO resection, VSD closure	None	Exertional dyspnea, perspiration	5 months of age	Diuretics
2	m	0.6	0.3	85–97	DCRV, VSD	Right sided aortic arch	RVOTO resection, VSD closure	None	Desaturation, tachypnea, rapid progression of RVOTO gradient	Neonatally	Beta-blocker
3	f	29.3	1.7	100	DCRV, spontaneous closure of former VSD	None	RVOTO resection	Surgical PDA closure	Exertional dyspnea	14 years of age	None
4	m	2.9	0.7	98	DCRV, VSD	None	RVOTO resection, VSD closure	None	Recurrent respiratory infections	Neonatally	Diuretics
5	f	0.4	0.3	96	DCRV, VSD	ASD II	RVOTO resection, VSD closure	None	None	Neonatally	Diuretics Beta-blocker
6	f	0.8	0.4	98	DCRV, VSD	None	RVOTO resection, VSD closure	None	Recurrent respiratory infections	Neonatally	None
7	m	7.5	1.1	99	DCRV, VSD	None	RVOTO resection, VSD closure	None	Perspiration, rapid progression of RVOTO gradient	Neonatally	None
8	m	0.3	0.3	99	DCRV, VSD	None	RVOTO resection, VSD closure	None	None	Neonatally	Beta-blocker
9	m	0.5	0.4	99	DCRV, VSD	PFO	RVOTO resection VSD closure	None	Asymptomatic, rapid progression of RVOTO gradient	Neonatally	None

*ASD* atrial septal defect, *BSA* body surface area (Du Bois method), *DCRV* double-chambered right ventricle, *m/f* male/female, *LAD* left anterior descending artery, *L/RVOTO* left/right ventricular outflow tract obstruction, *RCA* right coronary artery, *PFO* patent foramen ovale, *VSD* ventricular septal defect

Diagnosis of DCRV was predominantly established in the neonatal period or shortly thereafter (90%) and only in one patient (patient #3) it was diagnosed at the age of fourteen. In this patient, a VSD which had been described before, had functionally closed spontaneously and was pinhole-sized at the time of surgery, thus, initially, a VSD was present in all patients. Additional cardiac malformations were present in four patients. Patient #1 showed right and left ventricular outflow tract obstruction. Except for the closure of a patent ductus arteriosus through a lateral thoracotomy in patient #3, no prior procedures had been performed in this patient cohort.

Symptoms were variable from exertional dyspnea, perspiration, reoccurring respiratory infections and desaturation. Three patients (33.3%) were asymptomatic prior to surgery. In one out of these and in two additional patients with symptoms, significant progression of peak gradients measured across the RVOT was the main indication for surgery. More than half (5/9) of the patients were treated with beta-blocker and/or diuretics preoperatively. Mean follow-up of the entire cohort was very short with 1.6 ± 3.2 months.

### Echocardiographic Diagnosis and Assessment of Flow Disturbances

**Fig. 1:**
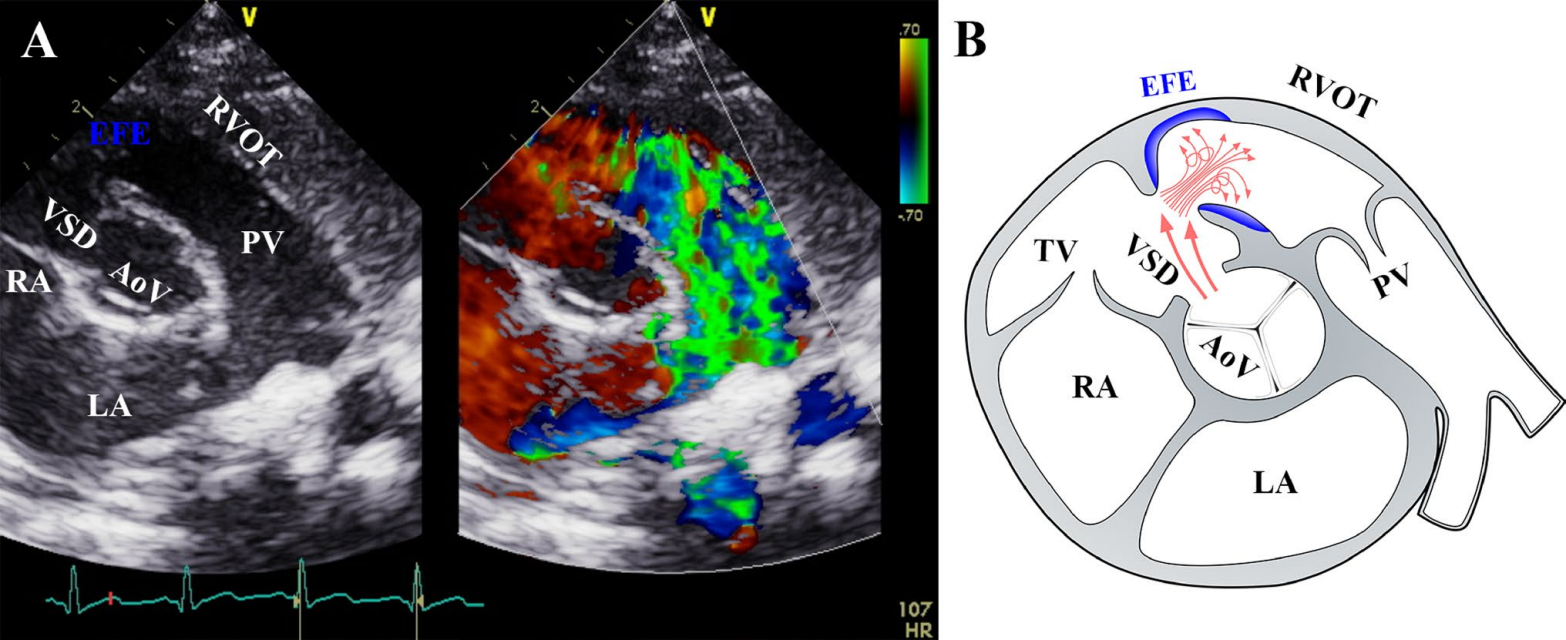
**A** Representative preoperative color-Doppler echocardiogram from a parasternal short-axis view. Diagnosis of DCRV is established via identification of key components (double-chambered RVOTO and VSD with significant flow disturbances) by echocardiography. A representative image is provided. **B** Sketch of (**A**): The associated graphical display of the DCRV pathology indicates the flow turbulence across the anomalous muscle bundles and the associated high-velocity turbulent flow jet. *EFE* endocardial fibroelastosis, *VSD* ventricular septal defect, *RVOT* right ventricular outflow tract, *PV* pulmonary valve, *AoV* aortic valve, *LA* left atrium, *RA* right atrium

At the time of surgery, a large non-restrictive VSD was present in all but one patient (Table 2). Localization was mostly subaortic (88.9%; 8/9). Malalignment was present in five patients (62.5%; 5/8). Shunt direction was left-to-right in the majority of patients (7/8), with only one patient having significant right-to-left shunting and suffering from cyanosis (patient #2). The median peak gradient across the VSD was 40 mmHg (IQR 7.3–72.5) and all patients displayed a turbulent high-velocity jet across the RVOTO (Fig. 1). Preoperative median peak flow velocity and peak gradient across the RVOT were 4.2 m/s (IQR 1.9–4.7) and 77 mmHg (IQR 55–91.5), respectively. Three patients presented with rapid progression of peak pressure gradients across the RVOT. Patient #2 showed a peak pressure gradient across the RVOT of 20 mmHg at birth, which gradually increased to 81 mmHg at 4 months of age. It remained at this level until surgery was performed at 8 months of age. Pulmonary valve (PV) morphology and diameter were normal in all patients. Postoperatively, the median measured peak RVOT gradient was 6 mmHg (IQR 4.5–17). No residual VSD was observed at the time of discharge. During short-term follow-up, no reoccurrence of RVOTO was documented in any patient (Table 2).

**Table 2:** Preoperative and postoperative echocardiography of the patient cohort

Patient	VSD morphology	Preop. max. gradient across VSD	Shunt direction across VSD	Preop. Vmax across RVOT	Preop. peak gradient across RVOTO	Preop. PV pathology	Postop. peak gradient across RVOT	Residual VSD shunt
1	Subaortic (∅ 8 mm), malalignment	45	L–R	1.2	77	None	12	None
2	Subaortic (∅ 7 mm), malalignment	3	R–L	4.5	82	None	5	None
3	Subaortic (almost closed) no malalignment	–	No shunt	1.2	88	None	30	None
4	Inlet (∅ 1.5 cm, partly covered)	75	L–R	4.3	75	None	10	None
5	Subaortic (∅ 8 mm, partly covered) no malalignment	35	L–R	3.3	44	None	22	None
6	Subaortic (∅ 8 mm, partly covered), malalignment	65	L–R	4.2	65	None	5	None
7	Subaortic (∅ 3 mm), no malalignment	4	L–R	4.9	95	None	3	None
8	Subaortic (∅ 5 mm), malalignment	92	L–R	2.6	45	None	4	None
9	Subaortic (∅ 8 mm) malalignment	17	L–R	4.9	97	None	6	Insignificant residual defect

*max. gradient* (in mmHg) systolic gradient across VSD/RVOT/PV, *RVOTO* sight ventricular outflow tract obstruction, shunt direction: *L-R/R-L* left-to-right/right-to-left shunt, *postop.* postoperative, *preop.* preoperative, *PR* pulmonary valve regurgitation, *PV* pulmonary valve, *Vmax* maximum flow velocity (in m/s), *VSD* ventricular septal defect

### Histological Analysis of Resected RVOT Tissue

**Fig. 2:**
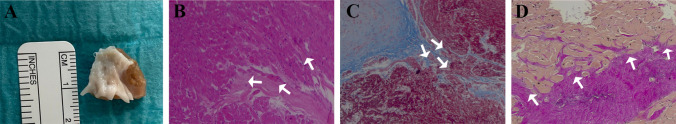
A representative RV tissue sample is shown which is comprised of muscle and fibrotic tissue (**A**). Histological analysis was performed through staining with hematoxylin and eosin (**B**) for assessment of overall tissue texture; with Masson’s Trichrome (**C**) for determination of fibrosis and Van Giesson (**D**) staining for visualization of elastin content. Collagen and elastin are prominently displayed subendocardially in organized layers and are also found infiltrating into the underlying myocardium (white arrows)

The resected tissue was localized in the areas depicted in the echocardiographic image and the corresponding sketch in Fig. 1. In all patients, the resected tissue samples contained muscle covered by a thick whitish layer on the endocardial facing surface as seen in Fig. 2A. In the histological analysis of H&E stainings, the overall texture of the myocardium was maintained but the myocardium was covered by thick layers of tissue with low cell counts (Fig. 2B). In several areas, this surface tissue showed growth patterns into the underlying myocardium. MT staining was used to identify collagen distribution in these samples. As shown in Fig. 2C, collagen indicated in blue was the predominant component of the thick whitish layer covering the myocardium with infiltration into the underlying myocardium in several areas. Furthermore, collagen and elastin surrounded the myocardial bundles diffusely throughout the myocardium. Subendocardial and intramyocardial collagen was accompanied by elastin. Collagen and elastin appeared in well-organized layers in the subendocardial space (Fig. 2C and D).

**Fig. 3:**
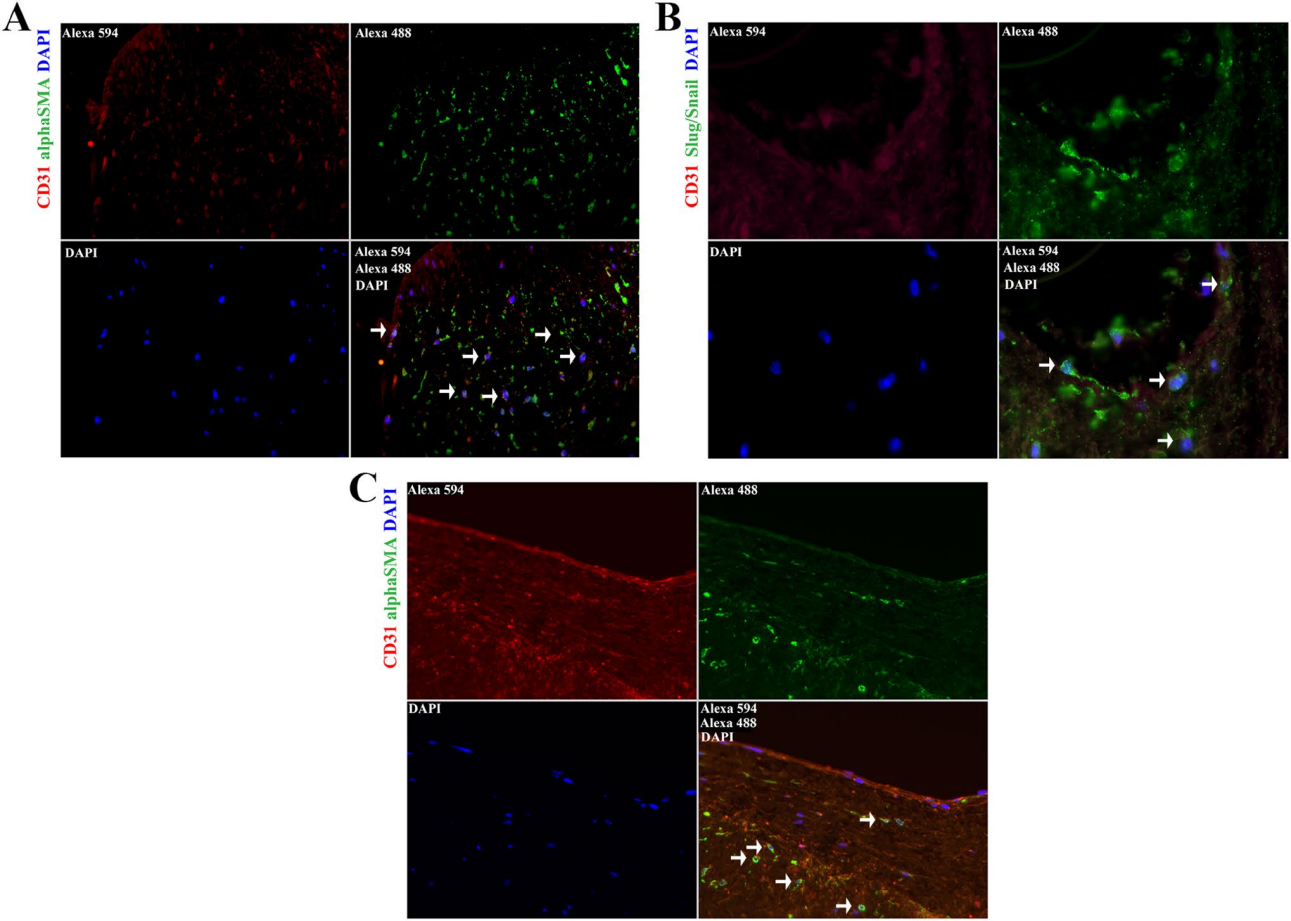
**A** Representative immunohistochemical stainings are shown. EndMT was verified by double-staining of endocardial endothelial cells with CD31 (in red) and αSMA (in green). Yellow colored cells are indicative of active EndMT as pointed out by white arrows. Nuclei appear in blue. As indicated, layers on top of the myocardium as well as areas of infiltrative growth into the myocardium are positive for EndMT (see white arrows) but the underlying myocardium is free from EndMT, except for one patient where the subendocardial myocardium shows a small number of EndMT positive endothelial cells. **B** Nuclei in CD31 positive cells double-stained for Slug/Snail are indicative of active EndMT (white arrows). CD31 is shown in red, nuclei stained with DAPI in blue and Slug/Snail in green. **C** Intramyocardial EndMT through double-staining of CD31 and αSMA (white arrow) as identified in one patient

As indicated in Fig. 3, all patients showed areas of double-stained cells (CD31/α-SMA) within the subendocardial layers but also within the areas which grew infiltratively into the underlying myocardium indicative for EndMT. The presence of active EndMT was confirmed by co-localization of stained endothelial cell nuclei with the transcription factors Slug/Snail (Fig. 3B). In all patients, subendocardial EndMT with connected areas of myocardial infiltrations were identified. In a single patient, capillary EndMT within the myocardium was also present (Fig. 3C).

**Fig. 4:**
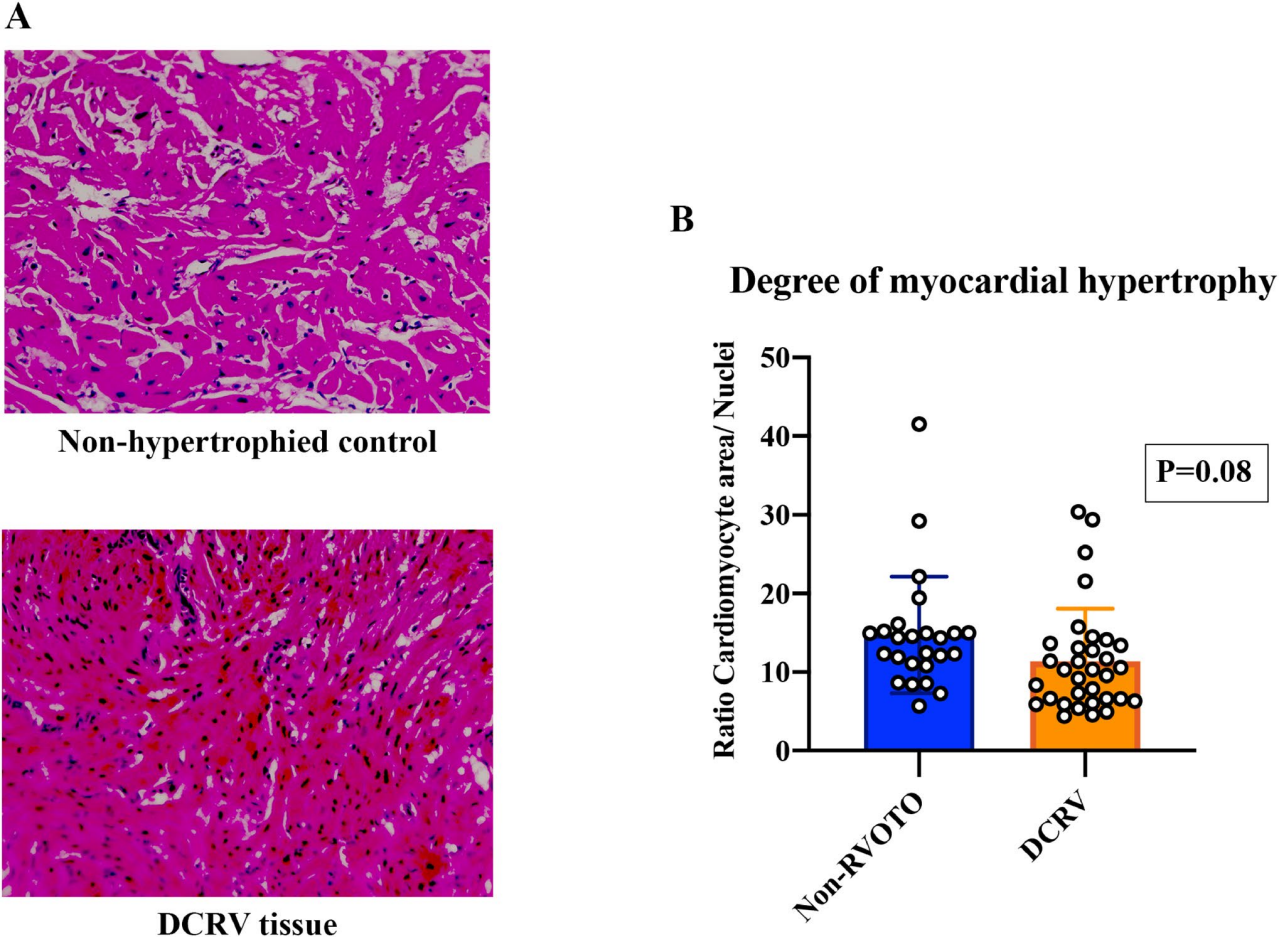
**A** Myocardial hypertrophy was determined by comparing the ratio of myocardium to number of nuclei per field of vision between DCRV tissue and non-hypertrophied human histological sections. **B** Summary statistics shows that there was no difference in the myocardial area/number nuclei ratio between the two groups. Data are expressed as mean ± SD

The degree of myocardial hypertrophy was not significantly different (*P* = 0.08) between DCRV samples and non-hypertrophied control samples (Fig. 4A and B).

## Discussion

In this study we set out to determine the remodeling process and associated triggers for the septation within the RV creating two different pressured chambers, better known as DCRV. All patients presented with flow disturbances across abnormal muscle bundles associated with the obstruction of the RVOT and across the VSD. Histological analysis of the tissue resected from the RVOTO during DCRV correction revealed that hypertrophy of the myocardium was not the only component of the formation of this septation, but the resected tissue resembled fibroelastic tissue with distinct layering of collagen and elastic fibers with infiltrative growth into the underlying myocardium. This fibroelastic tissue developed through localized activation of EndMT which was confirmed through immunohistochemical double-staining of endothelial cells by endothelial (CD31) and mesenchymal markers (α-SMA) and by activation of EndMT modulating transcription factors Slug/Snail. EndMT was, however, only associated with the endocardial endothelial cell layer but did not affect the capillary endothelial cells within the myocardium, except a few EndMT positive endothelial cells in the subendocardial myocardium in one patient, which is typically associated with myocardial hypertrophy as we have previously shown [12, 13]. Thus, DCRV is not only a result of hypertrophying muscle bundles but also due to an invasive remodeling process of the endocardium and underlying myocardium due to activation of EndMT.

Diagnosis of DCVR was performed by echocardiography in all cases. The presence of a clear septation within the RV into two separate chambers and a gradient across the outflow tract with significant turbulence without involvement of the pulmonary valve served as the basis to distinguish DCRV from Tetralogy of Fallot. Shortness of breath and exercise intolerance such as dyspnea on exertion have been described as the most common clinical symptoms which we confirmed in our patient cohort as well [14, 15]. Unless treated surgically, DCRV is a progressive disease with gradually increasing severity of the obstruction through the septation within the RVOT. Surgery is generally performed during childhood. Due to late referral of some out-of-the country patients, our patient cohort also included an adult patient which provides an interesting window into the natural progression of the disease. Most patients present with typical symptoms but the degree of incapacitation is highly variable which is associated with the wide range in severity of the RV obstruction. Especially older patients had only mild symptoms despite significant gradients across the RV which is in accordance with a study by Kahr et al. [4]. Rapid progression of the RVOT obstruction with increasing gradients, was the primary cause for referral in these patients. In the remaining patients of our cohort, indications for surgical correction were respiratory symptoms, indicative of worsening gradients across the RVOT. Resection of the obstructive lesion through surgery is the treatment of choice for symptomatic patients and/or patients with gradients greater than 40 mmHg [2]. Following successful resection to remove the RVOTO, long-term outcome is excellent. Re-operation for recurrent RVOTO was not necessary during a median follow-up of 8 years according to a previous study [4]. Long-term follow-up for our patient cohort is not yet available but repair of the underlying cause for flow turbulence may prevent localized remodeling through EndMT.

There is no consensus on the pathogenetic components that result in the development of DCRV. Hypertrophy of accessory or abnormal septo-parietal trabeculations associated with the moderator band have long been discussed as the underlying cause of DCRV formation [16]. However, Alva et al. characterized DCRV as a primary infundibular stenosis with obstructive fibrous muscle bands at the junction of the main RV cavity and the proximal infundibulum [17]. Muscle bundles as substrate for DCRV are potentially present at birth already but develop into an obstruction progressively over time due to localized alterations [18]. The results of our study implicate active EndMT with remodeling of the endocardium and subendocardial myocardium as a potential cause of such progressive obstruction.

As described by Nikolic et al., DCRV progression is associated with left-to-right shunting through a VSD which creates a jet directed toward the RV-free wall or crista supraventricularis, suggesting that flow turbulences might be associated with progressive obstruction [19]. In the majority of reported cases, a VSD is present which was also true for our patient population. In accordance with a report by Hubail et al., a subaortic VSD communicating with the proximal low-pressure chamber was the predominant defect. The reported intracavitary pressure difference of 67 mmHg to 83 mmHg within the RV was also similar to our findings [20].

As indicated by our data, myocardial hypertrophy of the anomalous muscle bundles projecting into the RV and obstructing blood flow within the RVOT is likely not the only element of progressive obstruction and DCRV formation. In fact, we documented fibrotic and elastic alteration of the septation similar to endocardial fibroelastosis. Organized layers of collagen and elastin as we have previously described in EFE tissue obtained from left ventricular diseases were predominantly present in these tissue samples [8, 9]. Furthermore, the fibrotic remodeling process infiltrated into the underlying myocardium which we have also seen in disease progression on the left side of the heart [8, 9]. The location of the VSD directly influences the hemodynamic profile since the VSD potentially stimulate localized remodeling which further aggravates the progression of the obstruction in the affected area and in turn results in additional high-velocity flow turbulences. The latter then trigger localized changes and stimulate cellular events such as EndMT of the endocardium leading to fibrotic remodeling of the outflow tract. These findings are similar to our previously published observations where flow turbulences with jets across stenotic valves on LV resulted in endocardial fibroelastosis formation. As indicated in Fig. 1, a high-powered jet and flow turbulences are visible which targets the area of the septation causing DCRV. Similar findings of jets and flow turbulences were described in an MRI study by Ibrahim et al. [21]. Rapid progression of the intraventricular gradient has been reported to result from a combination of increased localized myocardial hypertrophy and fibrosis. However, our study now established for the first time that this fibrotic process is the result of localized remodeling due to EndMT of the endocardium and may play a key role in the progression toward a DCRV [1]. Due to the small sample size, any conclusion regarding age differences in disease progression or degree of hypertrophy is not possible and has to be addressed in follow-up studies.

In conclusion, the initial septation of the RV is most likely due to abnormal muscle bundles as it has been widely discussed [20]. However, our results indicate that the ultimate progressive septation is a consequence of EndMT-induced fibrosis most likely triggered by flow disturbances imposed on the RVOT by blood flow turbulences caused by abnormal muscle bundles and across the VSD. Our current findings establish an interesting association which requires further research to establish a clear causal relationship and the tempo-spatial patterns of events leading to DCRV formation which may allow for early non-surgical intervention.

## Abbreviations

CD31: Cluster of differentiation 31
DCRV: Double-chambered right ventricle
EndMT: Endothelial-to-mesenchymal transition
H&E: Hematoxylin and eosin
IQR: Interquartile range
MT: Masson’s trichrome
PV: Pulmonary valve
RV: Right ventricle
RVOT/O: Right ventricle outflow tract/obstruction
α-SMA: α-Smooth muscle actin
VSD: Ventricular septal defect
